# A Case of Strange Worm Infection in a 21 Months Old Female in Karaj, Alborz Province, Iran

**Published:** 2019-02

**Authors:** Amir BAIRAMI, Enayatollah KALANTAR, Mohammad Hossein DEHGHAN, Monireh SEZAVAR, Parviz FALLAH, Aliehsan HEIDARI

**Affiliations:** 1. Department of Medical Laboratory Sciences, School of Allied Medicine, Alborz University of Medical Sciences, Karaj, Iran; 2. Rajaei Hospital, Alborz University of Medical Sciences, Karaj, Iran; 3. Department of Medical Parasitology, School of Medicine, Alborz University of Medical Sciences, Karaj, Iran

## Dear Editor-in-Chief

Helminthic infections are one of the commonest infections in man which affect a large proportion of the world’s population. They pose a major threat to public health particularly in developing countries (1). Luminal parasites have high morbidity all over the world (2). Helminths are among the major contributors to the global intestinal disease burden. Soil and water are of main routes of transmission of infections with parasitic worms and also accidental infections by non-parasitic worms like earthworms known as soil-transmitted helminths (STH). These infections are highest in communities with low hygienic condition but also take place in developed countries in susceptible persons (3). Infections by earthworms an uncommon parasitic worms that infect humans have been reported rarely (4). This article reports on a rare case of infection with a kind of earthworm diagnosed by optical microscopy in an Iranian 21-months-old female from Karaj.

**Fig. 1:**
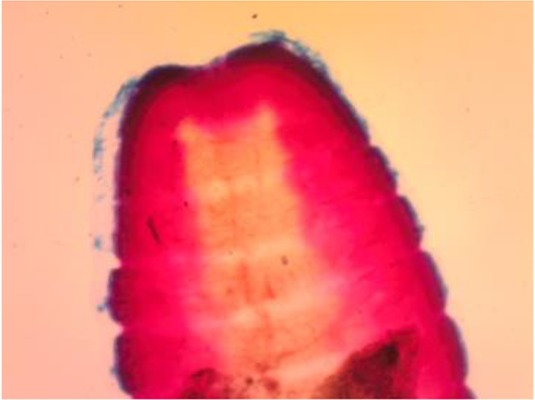
Worm in a microscopic examination

A 21-months-old female patient presented to the Emergency Department of Rajaei Hospital, Karaj, on Jul 9, 2017, with complaints of Stomach ache, Crowd, Nausea, Diarrhea, and fever 39 °C. Her weight was 10.5 kg. At the time of admission, according to the doctor suggested, stool examination was prescribed. In a microscopic examination (Fig. 1), a worm was found which 4 cm was and at first glance, the diagnosis of *Hymenolepis nana* was reported.

**Fig. 2:**
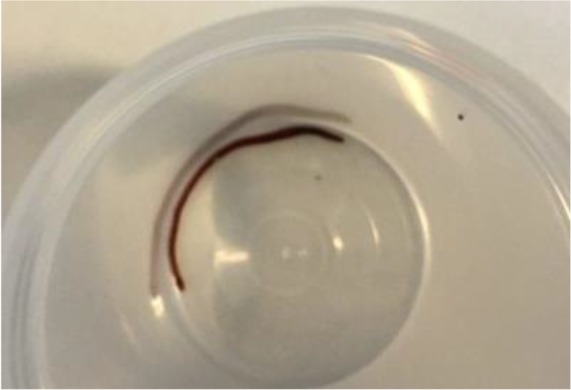
The whole worm in the plastic glass

However, after discussion with a quite a few medical parasitologists based on the wrapping of the worm, its size, and hooks under microscope, we finally reported an unusual earthworm. The worm in the plastic glass is shown in Fig. 2.

According to the initial diagnosis, treatment with niclosamide started. Interestingly, after the treatment, the symptoms of the baby were resolved and the second stool examination was negative. Based on interview with the baby’s parents, they have been out of station and their economic status was not in a sound position; they usually went to north of the country and their baby contacts directly with soil and water. Moreover, both the parents were also stool examined and they were negative for eggs or larvae of the parasite.

Parasitic worms are one of the most indictable infections influence mankind. Exact prevalence of gastrointestinal parasites in Iran and even other countries are almost impossible to obtain. The vast majority of research and literature has concentrated on areas of high prevalence including Asia, South America, and Africa (5); although, gastrointestinal parasite infections are most prevalent in areas of poverty, in low and middle-income countries (6); however, they still affect many countries. The global target is to eliminate morbidity due to STH in children by 2020 (3). Earthworms as a worldwide distribution and astoundingly are not the pathogenic members of the worms. To our knowledge, previously, there were rare cases of human infection with earthworms have been reported in the world (4).

There should be a high suspicion for parasitic infestation in babies with sudden acute intestinal pain to prevent life-threatening complications.
